# Blood culture diagnostics - a comparative and experimental study on the impact of delayed incubation

**DOI:** 10.1186/s12866-025-04623-y

**Published:** 2026-01-09

**Authors:** Andreas Jacobs Østerhegn Jensen, Louise Thomsen, Torgny Sunnerhagen, Christian Johann Lerche, Claus Moser

**Affiliations:** 1 Department of Clinical Microbiology, Copenhagen University Hospital, Rigshospitalet, Copenhagen, Denmark; 2 University College Copenhagen, Copenhagen, Denmark; 3 Clinical Microbiology, Office for Medical Services, Region Skåne, Lund, Sweden; 4 Division of Infection Medicine, Department of Clinical Sciences Lund, Lund University, Lund, Sweden; 5 Department of Immunology and Microbiology, University of Copenhagen, Copenhagen, Denmark

**Keywords:** Automated blood culture systems, Blood culture diagnostics, Bloodstream infection, Collection-to-detection time, Delayed incubation, Diagnostic workflow

## Abstract

**Objectives:**

This study investigates the impact of transitioning from restricted to 24/7 access to blood culture cabinets on blood culture processing.

**Method:**

A post-hoc retrospective study and a prospective laboratory simulation to evaluate the effects of delayed incubation were conducted. Data analysis evaluated clinical data comparing incubation-to-detection (ITD) and Collection-to-detection (CTD) including Collection-to-incubation (CTI) comparing pre- and post-implementing the new protocol (Cut-point). ITD values were obtained using BD Synapsys™ software, with delays factored into CTD. Of 14,673 blood cultures collected from October 2019 to September 2023 at Rigshospitalet, Copenhagen, 3,323 met inclusion criteria. *Escherichia coli*,* Staphylococcus aureus*,* Pseudomonas aeruginosa* and *Streptococcus pneumoniae* were selected as indicator organisms. In the simulation, cultures with 0-, 6-, and 18-hour incubation delays were assessed for growth and detection time.

**Results:**

Median ITD increased significantly post-cut-point: for *E. coli* from 7.8(IQR: 2.28–13.55) to 11.4(IQR: 9.8–14.7) hours, *S. aureus* from 9.9(IQR: 4.1–15.6) to 14.3 h(IQR: 11.5–19.5), *P. aeruginosa* from 14.0(IQR 5.3-19.95) to 16.3 h(IQR 11.75–26.1), and *S. pneumoniae* from 3.3(IQR: 1.55–9.9) to 11.7 h(IQR: 9–12.6) (all *p* < 0.01). CTD decreased post-cut-point: by 3h03m for *E. coli* (*p* < 0.0001), 3h51m for *S. aureus* (*p* = 0.0016), and 4h24m for *S. pneumoniae* (*p* < 0.0001). The reduction for *P. aeruginosa* (1h48m) was not statistically significant.

In the simulation-study, delayed incubation increased CTD for all species in aerobic bottles: *E. coli* (p = 0.0036), *S. aureus* (p = 0.0036), *P. aeruginosa* (p = 0.0036), and *S. pneumoniae* (p = 0.0429); and in anaerobic bottles: *E. coli* (p = 0.0036), *S. aureus* (p = 0.0036), and *S. pneumoniae* (p = 0.0071). No anaerobic growth of *P. aeruginosa* was observed. An 18-hour delay notably reduced recovery of *S. pneumoniae*, with growth detected in only one bottle.

**Conclusion:**

Minimizing incubation delays significantly reduces CTD and improves detection of fragile bacteria. These findings potentially have significant implications for clinical practice, emphasizing the importance of protocols that limit pre-analytical delays to optimize blood culture diagnostics.

## Importance

Bloodstream infections are life-threatening and require rapid, accurate diagnosis to guide treatment. However, delays in processing blood cultures are common in clinical settings, potentially impacting patient outcomes. This study examines how delayed incubation affects the detection of bloodstream infections on the Collection-to-detection (CTD), combining statistical data analysis with experimental laboratory work. Our findings provide critical insights that could improve laboratory protocols to ensure faster more reliable identification and recovery of fragile pathogens. By optimizing blood culture diagnostics, healthcare providers can make better-informed treatment decisions, ultimately saving lives.

## Introduction

Blood culturing is essential and gold standard for diagnosing bloodstream infections (BSIs) [1, 2], conditions associated with high morbidity, mortality, and frequent need for intensive care [1–5]. Timely and accurate diagnosis of BSIs through blood culture is vital for initiating appropriate antimicrobial therapy, improving clinical outcomes, and reducing healthcare costs [6–9]. Although many studies focus on incubation-to-detection (ITD) interval, the total collection-to-detection (CTD) time - which includes pre-analytical delays - is less well characterized, despite its direct clinical relevance. Evidence suggest that reducing pre-incubation delays through continuous loading of blood culture bottles (BCBs) can shorten time to detection and accelerate diagnostic workflows [10].

Clinical guidelines recommend incubating BCBs within two hours of collection, to preserve pathogen viability and minimize false-negative results, particularly for fastidious organisms [11]. However, in many clinical settings, delays are not uncommon due to transportation logistics, limited laboratory operating hours, and workflow constraints. Such delays may reduce recovery of fastidious pathogens, prolong time to positivity, and subsequently delay antimicrobial susceptibility testing and the transition from empirical to targeted therapy. In rare cases, prolonged delays may even shift the microbial composition within a bottle, allowing more resilient species to outcompete sensitive ones [12, 13]. While continuous 24-hour incubation services are increasingly implemented, it is important to note that transport-related delays may still occur.

The setup for blood culture diagnostics was changed at our referral university hospital to minimize pre-incubation delays. Previously, blood cultures collected outside the Department of Clinical Microbiology (DCM) working hours (7:00–15:00) frequently experienced extended pre-incubation times. To address this, a new procedure was implemented on the 28th of September 2021 (the “cut-point”), in collaboration with the Department of Clinical Biochemistry (DCB), which operates around the clock. Blood culture incubators were installed within DCB, allowing immediate loading regardless of the time. Although operationally more resource-intensive, this change was expected to improve diagnostic timeliness and quality.

We hypothesized that implementing continuous 24/7 incubation would reduce CTD primarily by shortening collection-to-incubation (CTI) interval, even if the relative contribution of the ITD phase was altered. The primary aim of this study was to how delayed incubation affects CTD, ITD, and organism recovery using both retrospective clinical data and controlled laboratory simulation experiments. Specifically, we examined whether delay-associated effects observed in laboratory models translate to routine clinical practice and whether organisms respond similarly to pre-incubation delays across experimental and real-world settings.

## Method

### Study design

This study was conducted at the Department of Clinical Microbiology (DCM), Copenhagen University Hospital, Rigshospitalet, a 1038-bed tertiary referral hospital.

The study was structured in two components:

1. *A retrospective observational study* analyzing routinely collected clinical blood culture data before and after a logistical intervention that reduced incubation delays.
2. *A prospective in vitro simulation study* modeling delayed incubation of spiked blood culture bottles with selected bacterial species under controlled laboratory conditions.

Both components of the study assessed the impact of delayed incubation on two key intervals:

- *Incubation-to-detection (ITD)*: Time from the start of incubation to the automated detection of microbial growth, reflecting only the incubation phase.
- *Collection-to-Detection (CTD)*: Total time from blood collection to detection of microbial growth, incorporating both the pre-incubation delay (Collection-to-Incubation Time, CTI) and the incubation phase (ITD).

By comparing ITD and CTD under minimal-delay versus delayed-incubation conditions, the study evaluated how pre-incubation delays influence diagnostic timelines in both clinical and experimental settings.

Blood cultures are incubated for up to 7 days before being reported negative, in accordance with institutional guidelines.

During the retrospective and simulation studies blood culture samples were processed using the BACTEC FX automated blood culture system (Becton Dickinson, BD, East Rutherford, NJ, USA). Aerobic samples were collected in BACTEC™ Plus Aerobic bottles, and anaerobic samples in BACTEC™ Lytic Anaerobic bottles. No changes to the incubation platform or bottle types occurred during the study period.

## Retrospective clinical data study

### Data source and extraction

The data analysis utilized archived clinical data on blood cultures collected over a four-year period from October 2019 to end September 2023 from the laboratory information system MADS (Mikrobiologisk Afdeling Data System) which captures all blood culture results and associated timestamps. The dataset included all blood cultures processed within two years before and two years after the date of September 28th, 2021 (Cut-point).

A total of 14.672 blood culture samples were identified. We chose four of the most prevalent pathogens causing BSI: *Escherichia coli*, *Staphylococcus aureus*, *Pseudomonas aeruginosa*, and *Streptococcus pneumoniae*. Based on organism identification and data completeness, 3323 samples were included in the analysis. No algorithmic tools or machine learning were used; all microbial identifications were based on final laboratory-confirmed results. No external data linkages were performed.

Data access was restricted to authorized study personnel, and all data were handled in accordance with institutional data protection guidelines. The study was designed and reported in accordance with the RECORD guidelines [14]. 

### Blood culture timing definitions

Time intervals in this study are defined as follows (summarized in Table 1):

**Table 1:** Blood culture timing terminology

Abbreviation	Full Term	Definition	Notes
CTD	Collection-to-Detection	Time from blood sample collection to positive detection of bacterial growth in the blood culture system	Overall diagnostic turnaround time; reported in hours (h)
CTI	Collection-to-Incubation	Time from blood sample collection to start of incubation	Includes transport and pre-incubation handling; reported in hours (h)
ITD	Incubation-to-Detection	Time from start of incubation to positive detection of bacterial growth	Reflects intrinsic microbial growth and detection kinetics; reported in hours (h)

All times are reported in hours (h)

## Sample selection

Only blood culture samples with a positive identification of *E. coli*,* S. aureus P. aeruginosa* or *S. pneumoniae* were included. Samples were excluded if they lacked relevant information such as timestamps (collection, incubation start, or detection), contained non-blood specimens, or had incomplete laboratory information. Specifically, of the initial 1385 *E. coli* samples, 115 were excluded (final *n* = 1270); for *S. aureus*, 1962 samples were included and 157 excluded (final *n* = 1805); for *P. aeruginosa*, 81 of the 227 total samples were excluded (final *n* = 146); and all 102 *S. pneumoniae* samples were retained.

### Filtering samples and processing CTD time

The clinical samples were initially categorized by bacterial species. Subsequently, they were subdivided into two groups relative to the Cut-point (Table 2). 

**Table 2:** Incubation-to-detection (ITD)

	Samples		Median / IQR (h)		Median diff (95% CI)	*p*-value
Bacterial species Total samples, (*n*) (*n* = 3323)	Before Cut-point	After Cut-point	Before Cut-point	After Cut-point		
*Escherichia coli* *Total n (%) = 1270 (38%)*	589 (46.38%)	681 (53.62%)	7.8 (IQR 2.28–13.55)	11.4 (IQR 9.8–14.7)	3.6 (95% CI 2.4–4.8)	< 0.0001
*Staphylococcus aureus* *Total n (%) = 1805 (55%)*	895 (49.53%)	910 (50.47%)	9.9 (IQR 4.1–15.6)	14.3 (IQR 11.5–19.5)	4.4 (95% CI 3.0-5.9)	< 0.0001
*Pseudomonas aeruginosa* *Total n (%) = 146 (4%)*	69 (47.59%)	77 (52.41%)	14 (IQR 5.3-19.95)	16.3 (IQR 11.75–26.1)	2.3 (95% CI 0.7–4.1)	0.0034
*Streptococcus pneumoniae* *Total n (%) = 102 (3%)*	30 (29.41%)	72 (70.59%)	3.3 (IQR 1.55–9.9)	11.7 (IQR 9-12.6)	8.4 (95% CI 6.0-10.8)	< 0.0001

All clinical blood culture samples extracted included data on collection time and date, as well as the ITD time. Determining CTD required manual calculation by adding the CTI interval to the ITD interval. Because *E. coli* and *S. aureus* were highly prevalent, CTD calculation required manual verification of CTI and ITD for each episode. To maintain feasibility while preserving statistical power, a random sample of 100 bottles before and 100 after the Cut-point was selected for each of these species using the Google Sheets randomization function (Google LLC, Mountain View, CA, USA). For *P. aeruginosa* and *S. pneumoniae*, CTD was calculated for all available samples. A sample size of 100 per group, two-sided α = 0.05 and target power 80%, the minimal detectable standardized effect is Cohen’s d ≈ 0.40. Translating this to CTD units gives a detectable difference of approximately 1.2–4.4 h depending on the assumed CTD standard deviation (e.g., ≈ 2.0 h for SD = 5 h; ≈3.6 h for SD = 9 h).

### Ethics

This study was conducted as a quality assessment study. The analyses utilized anonymized clinical samples, ensuring that no patient identifying information was included. This approach endorsed patient privacy, aligning with ethical standards in research. Given that the samples were devoid of any personal identifiers, formal ethical approval was not required according to Danish law.

### Simulated blood culture study

The in vitro simulation study evaluated how pre-incubation delays of 0, 6, and 18 h affected ITD and CTD.

### Bacterial strains

For each species, one ATCC reference strain (*E. coli* ATCC25922, *S. aureus* ATCC29213, *P. aeruginosa* ATCC27853, *S. pneumoniae* ATCC49619) and two clinical bloodstream isolates were used. Clinical isolates were randomly selected from the institutional strain collection and originated from confirmed BSIs within the previous 12 months. All isolates were identified using MALDI-TOF (Bruker Daltonics, Bremen, Germany).

### Blood culture preparation

Aerobic and anaerobic BCBs (BD BACTEC™ Plus Aerobic medium and BD BACTEC™ Lytic Anaerobic medium, Becton Dickinson, East Rutherford, NJ, USA) were inoculated with bacterial-spiked horse blood (Håtunalab AB, Håtunaholm, Sweden) and incubated in the BD BACTEC™ FX Blood Culture System. A bacterial concentration of 14 CFU/mL, considered a relevant bacterial concentration in clinical samples, was used [2]. Sterile defibrinated horse blood served as the matrix.

Each pair of bottles consisted of:

- one incubated immediately (0-hour delay), and
- one held at room temperature (20–24 °C) for a 6-hour or 18-hour delay.

### Inoculum preparation

Frozen isolates (-80 °C) were sub-cultured on 5% blood agar (SSI Diagnostica, Hillerød, DK) and incubated overnight at 35 °C (± 2 °C). Colonies were suspended in 0.9% sterile saline (SSC Panum, Copenhagen, DK) to a 0.5 McFarland standard (1.5 × 10^8^ CFU/mL; Biosan, Riga, Latvia).

A series of dilutions (1:100 -> 1:100 -> 1:40) yielded a theoretical concentration of 375 CFU/mL. The final concentration was confirmed by plating 100 µL of the suspension onto three blood agar plates for colony counting. The mean colony count was used to calculate the true CFU/mL.

Two milliliters of the final suspension were mixed with 50 mL horse blood to obtain ~ 14 CFU/mL. Bottles were then filled with 10 mL of spiked blood.

### Statistical analysis

Data distributions were visually assessed and found to be right-skewed; thus, non-parametric methods were used.

### Retrospective study

Continuous variables were summarized as medians with interquartile ranges (IQRs). Group comparisons (Group A: before the Cut-point; Group B: after the Cut-point) for each bacterial species were performed using the Mann-Whitney U test. Effect sizes were expressed using the Hodges–Lehmann estimator with corresponding 95% confidence intervals.

Data visualization included boxplots showing medians, IQRs, and individual data points. Analyses were performed using GraphPad Prism (GraphPad Software, version 10, Inc., San Diego, USA).

### Simulation study

ITD values were obtained using BD Synapsys™ software (BD Biosciences, San Jose, CA, USA). For each species, mean ITD values across strains were calculated, and delay times (0, 6, 18 h) were added to derive CTD estimates. Group comparisons across delay intervals were performed using the Kruskal–Wallis test for each species. P values < 0.05 was considered statistically significant.

## Results

### Retrospective study

#### Incubation-to-detection (ITD)

Across all four bacterial species, ITD increased significantly after the cut-point (Table 2). For *E. coli*, the median ITD increased from 7.8 h (IQR: 2.3–13.6 h) to 11.4 h (IQR: 9.8–14.7 h), median difference 3.6 h (95% CI: 2.4–4.8 h), *p* < 0.0001. For *S. aureus*, ITD increased from 9.9 h (IQR: 4.1–15.6 h) to 14.3 h (IQR: 11.5–19.5 h), median difference 4.4 h (95% CI: 3.0–5.9 h), *p* < 0.0001. *P. aeruginosa* showed an increase from 14 h (IQR: 5.3–20 h) to 16.3 h (IQR: 11.8–26.1 h), median difference 2.3 h (95% CI: 0.7–4.1 h), *p* = 0.0034. Finally, *S. pneumoniae* demonstrated an increase from 3.3 h (IQR: 1.6–9.9 h) to 11.7 h (IQR: 9–12.6 h), median difference 8.4 h (95% CI: 6.0–10.8 h), *p* < 0.0001.

These increases likely reflect workflow-related factors such as diurnal variation and timing of overnight blood culture loading rather than true biological differences in bacterial growth kinetics.

### Clinical Collection-to-detection (CTD)

**Fig. 1:**
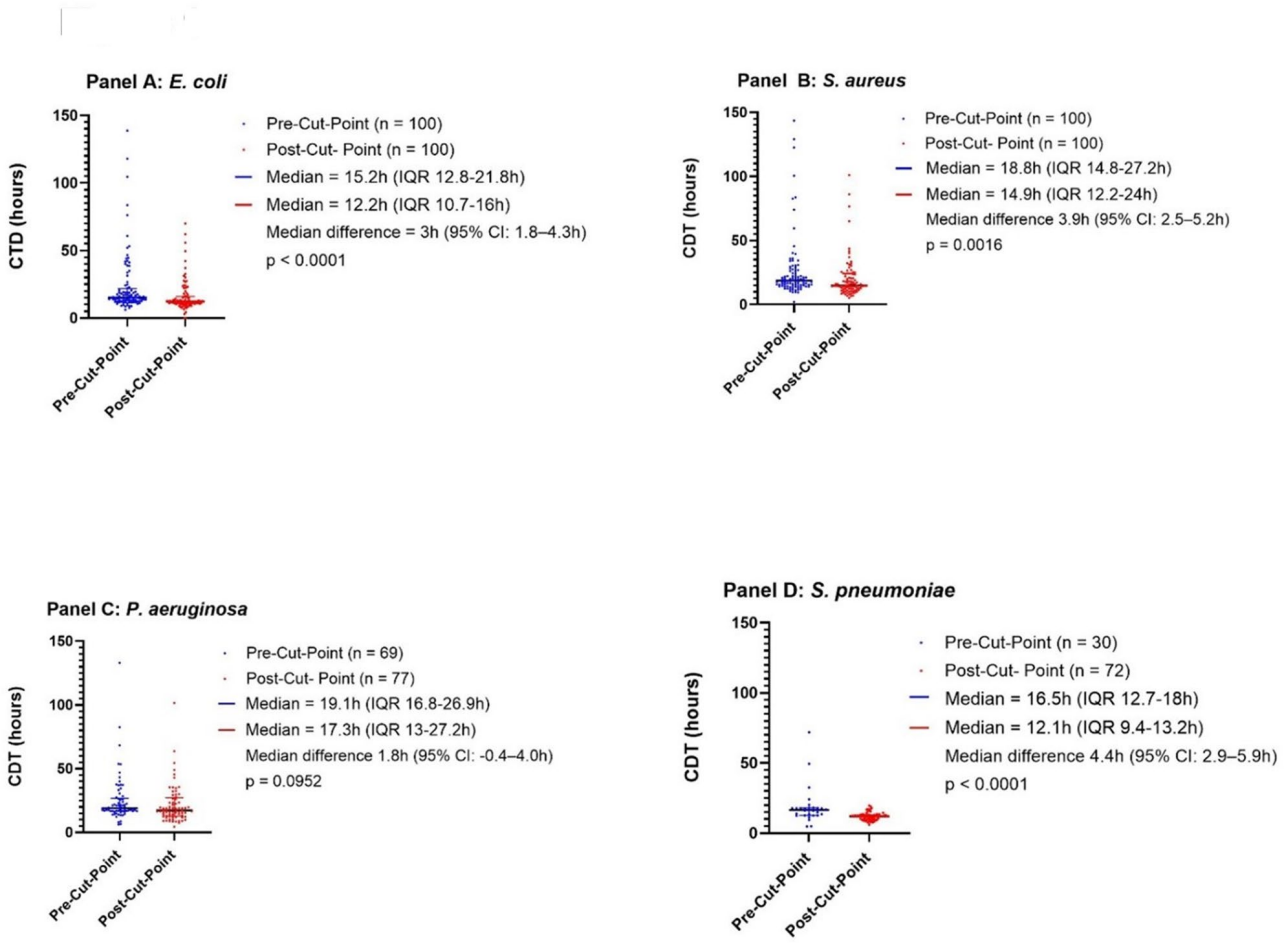
Collection-to-detection (CTD) times before and after implementation of continuous (24/7) incubation. Each point represents an individual blood culture episode, calculated as incubation-to-detection (ITD) time plus collection-to-incubation (CTI) time. Median CTD values with interquartile ranges are shown. Panel **A**: E. coli (pre-cut-point: *n* = 100; post-cut-point: *n* = 100). Panel **B**: S. aureus (pre-cut-point: *n* = 100; post-cut-point: *n* = 100). Panel **C**: P. aeruginosa (pre-cut-point: *n* = 69; post-cut-point: *n* = 77). Panel **D**: S. pneumoniae (pre-cut-point: *n* = 30; post-cut-point: *n* = 72)

CTD decreased for all bacterial species after implementation of the 24/7 incubation workflow (Fig. 1). For *E. coli*, the median CTD decreased by 3 h and 3 min (to 12.15 h; IQR: 10.7–16 h), median difference 3 h (95% CI: 1.8–4.3 h). For *S. aureus*, CTD decreased by 3 h and 51 min (to 14.9 h; IQR: 12.2–24 h), median difference 3.9 h (95% CI: 2.5–5.2 h). *S. pneumoniae* showed a reduction of 4 h and 24 min (to 12.1 h; IQR: 9.4–13.2 h), median difference 4.4 h (95% CI: 2.9–5.9 h). For *P. aeruginosa*, CTD decreased by 1 h and 48 min (to 17.3 h; IQR: 13–27.2 h), although this did not reach statistical significance (median difference 1.8 h; 95% CI: − 0.4–4.0 h).

Overall, these results demonstrate that continuous 24/7 incubation effectively reduced pre-analytical delays (shorter CTI), leading to clinically meaningful reductions in CTD.

### Simulated blood culture study

#### Incubation-to-detection (ITD)

In the controlled simulation, all species showed shorter ITD when incubated without delay compared with delayed incubation. *S. pneumoniae* showed reduced recovery after delayed incubation: after a 6-hour delay, only one anaerobic bottle became positive; after an 18-hour delay, only one bottle (aerobic or anaerobic) showed any growth (Table 3). 

**Table 3:** ITD (Simulated study on blood cultures)

Bacterial species investigated (*n* = 3 strains / bacterial species)	Aerobic bottles Collection to incubation (Delay time to incubation in hours (h))			Anaerobic bottles Collection to incubation (Delay time to incubation in hours (h))		
*Hours*	(0)	(6)	(18)	(0)	(6)	(18)
*Escherichia coli* (*n* = 3)	11.09	7.92	4.22	10.33	7.53	3.96
*Staphylococcus aureus* (*n* = 3)	13.42	11.3	7.14	12.32	10.3	6.09
*Pseudomonas aeruginosa* (*n* = 3)	16.24	14.01	10.34			
*Streptococcus pneumoniae* (*n* = 3)	12.54	11.67	31	24.8	27.7	n.a

Median incubation-to-detection (ITD) times for each bacterial group in aerobic and anaerobic blood culture bottles under fast incubation (0-hour delay) and delayed incubation (6- and 18-hour delays). ITD represents the time from placement in the incubator to detection, excluding any pre-incubation delay. The total collection-to-detection (CTD) time includes the ITD plus the pre-incubation delay (6 or 18 hours)

*NA* not applicable, no growth detected under the specified delay condition. -ITD alone may appear shorter under longer delays in some experimental conditions due to early growth initiation during the pre-incubation period. CTD, which accounts for the full delay, increases as expected with longer incubation delays

#### Collection-to-detection (CTD)

**Fig. 2:**
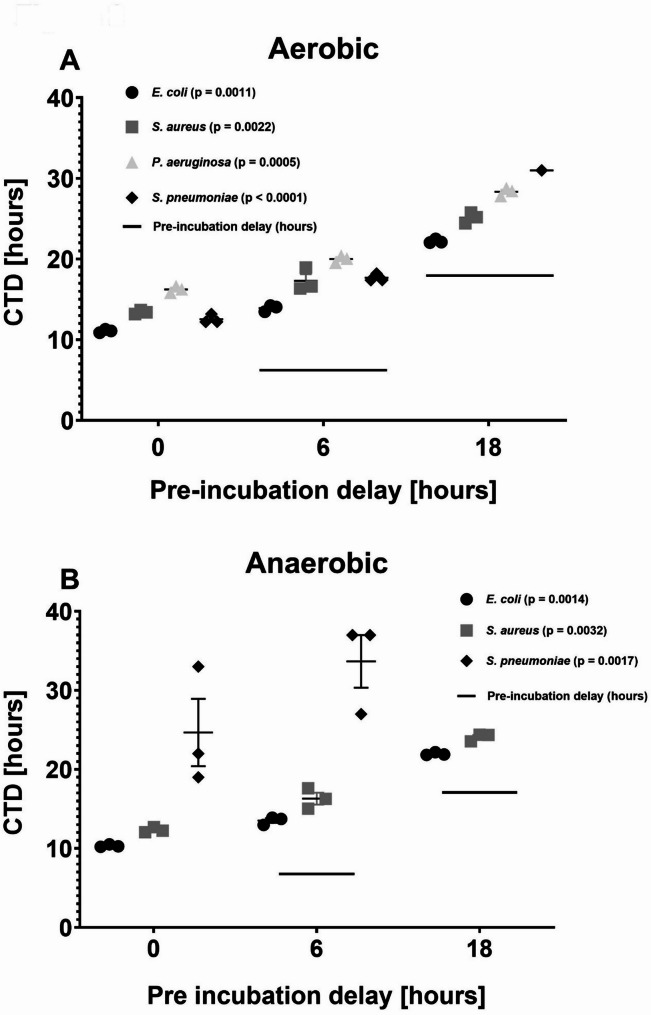
Collection-to-detection time (CTD) for *E. coli*, *S. aureus*, *P. aeruginosa*, and *S. pneumoniae* following 0, 6, and 18 h of delayed incubation. Panel **A** shows CTD in aerobic bottles, and Panel **B** shows CTD in anaerobic bottles. CTD increased significantly with longer delays for all species in aerobic bottles (Panel **A**), and for *E. coli*,* S. aureus*, and *S. pneumoniae* in anaerobic bottles (Panel **B**). P-values for all pairwise comparisons are shown. Note: The y-axis is inverted, with shorter CTD values appearing lower on the axis, to enhance visual comparison across delay conditions

Adding the pre-incubation delay (CTI) resulted in significantly prolonged CTD for all species under delayed incubation. In aerobic bottles, delayed incubation significantly increased CTD for *E. coli*, *S. aureus*, *P. aeruginosa*, and *S. pneumoniae* (*p* = 0.0036 for all except *S. pneumoniae*, *p* = 0.0429). The same pattern was observed for anaerobic bottles, with significantly prolonged CTD for *E. coli*, *S. aureus*, and *S. pneumoniae* (*p* = 0.0036, *p* = 0.0036, and *p* = 0.0071, respectively) (Fig. 2).

*P. aeruginosa* showed no growth in anaerobic bottles.

Although ITD increased after the cut-point in the clinical dataset, this likely reflects workflow-related timing differences (e.g., delayed loading and diurnal variation rather than changes in intrinsic growth behavior. The simulation study confirms, under controlled conditions, rapid incubation inherently shortens ITD and CTD.

## Discussion

The present study revealed that changing a tertiary referral hospital strategy to incubate blood culture bottles 24/7 significantly shortened the overall Collection-to-detection (CTD) time for the most common bloodstream infection pathogens. The simulation experiments supported these findings by showing that delayed incubation adversely affected organism viability, particularly for *Streptococcus pneumoniae*, an occurrence that could have clinical implications [15]. These results align with previous work indicating that continuous loading reduces CTD and enhances laboratory workflow efficiency [10]. Collectively, our findings emphasize the importance of optimizing both logistics and incubation procedures to accelerate the diagnosis of bloodstream infections.

The simulation experiments provided mechanistic insight into how delayed incubation influences bacterial viability. Consistent with earlier studies [10, 12, 16, 17], *S. pneumoniae* showed pronounced vulnerability to pre-incubation delays: recovery declined substantially after 6 h and was nearly lost after 18 h, likely related to autolysis and low inoculum stability [17–19]. Other fastidious or fragile organisms, including *Haemophilus influenzae*, *Neisseria gonorrhoeae*, *Legionella pneumophila*, are similarly sensitive to suboptimal handling and may be affected by comparable delay [20, 21].

In the retrospective dataset, we did not observe a decrease in the frequency of *S. pneumoniae* detections after the cut-point. This is likely because real-world pre-incubation delays rarely approached the prolonged delays used in the simulation experiments. In contrast, the experimental 18-hour delay produced substantially loss of viability, consistent with the organism’s high autolytic potential. Low inoculum volumes may further amplify this effect. Importantly, the clinical dataset inherently captures only cultures that eventually turned positive. Cultures in which bacteria were completely non-viable before incubation would appear as false negatives but cannot be retrospectively identified. Thus, while the simulation study demonstrates biological plausibility for delayed-incubation false negatives, the frequency of such events in practice cannot be determined from retrospective data.

The simulation also showed that ITD was shorter after delayed incubation; however, when the delay time was included, the overall CTD increased, underscoring the critical importance of timely incubation. Despite marked improvement in CTD following the introduction of continuous incubation, the clinical dataset showed a modest increase in ITD. Several operational factors may explain this observation. First, the 24/7 workflow increased the proportion of samples loaded during overnight hours, where differences in bacterial load or growth kinetics may modestly prolong ITD. Second, the transition from batch loading in the morning to continuous, single-sample loading throughout the day and night could influence how the instrument registers growth curves and triggers positivity. Third, minor instrument-related differences such as distributing incubation capacity across two departments or variations in instrument calibration schedules may contribute to subtle ITD changes without reflecting true differences in microbial growth. Importantly, this increase in ITD does not diminish the overall finding that CTD is primarily driven by pre-analytical delays (CTI), and the net effect continuous incubation is a clinically significant reduction in total detection time.

While many studies emphasize ITD as a performance metric, CTD more directly influences clinical decision-making. Prolonged CTD delays species identification, susceptibility testing, and transition from empirical to targeted therapy, increasing both the risk of ineffective treatment and unnecessary antimicrobial exposure [9, 16, 22–24]. Previous work has shown that reducing pre-incubation delays can accelerate clinical adjustments even if immediate effects on length of stay or mortality are limited [23, 24]. Despite strong recommendations for rapid incubation, compliance remains challenging, particularly during off-hours [25]. Addressing these logistical barriers is essential to achieve the full clinical benefit of optimized blood culture workflows.

This study did not evaluate downstream processes such as Gram-stain timing or reporting workflows, which may influence the clinical impact of earlier incubation. Temperature is another important variable: bottles in this study were kept at room temperature, whereas higher temperatures have been associated with increased false negatives in fastidious species [26]. Additionally, centralized laboratory models serving multiple hospitals often face longer transport times, further prolonging CTD [27]. These organizational factors warrant consideration when designing optimal blood culture pathways.

Precise interpretation of temporal patterns was also limited by inconsistencies in timestamp quality, which prevented stratification by time of collection (e.g., within versus outside regular laboratory hours). As noted in other retrospective workflow analyses [10], incomplete metadata on staff activity and protocol adherence introduces uncertainty into analyses of operational delays. Improved timestamp accuracy will be essential for more granular assessments in future research.

### Limitations

This study has several important limitations.

First, in the retrospective CTD analysis, only a subset of 200 *E. coli* and 200 *S. aureus* positive cultures (100 pre– and 100 post–cut-point) was analyzed due to the manual nature of timestamp verification. Although random selection minimizes bias, this relatively modest sample reduces statistical power and may limit generalizability. The sample size reflected a balance between analytical rigor and the substantial manual workload involved. Larger datasets or automated timestamp extraction methods would strengthen future analyses.

Second, the simulation experiments included a limited number of replicates, particularly for *S. pneumoniae* and *P. aeruginosa*. Reduced recovery of *S. pneumoniae* after delayed incubation limited available data. While this finding illustrates the potential for severe viability loss after prolonged pre-incubation delays, such delays were rare in the clinical workflow, which likely explains why the retrospective data did not show an increased proportion of missed positives after continuous incubation was implemented. Moreover, because retrospective clinical data only include cultures that became positive, true false negatives caused by loss of viability cannot be identified. These factors may mask clinically relevant but infrequent losses in recovery; thus, simulation findingss should be interpreted with caution, and larger controlled studies are warranted. Nevertheless, compared with previous simulation-based work such as Johnsson et al. [25], our study provides complementary insights by combining experimental and real-world clinical data to characterize organism-specific effects of delayed incubation.

Third, retrospective clinical data lacked information on collected blood volume and prior antibiotic therapy two variables known to affect culture sensitivity and detection time. While institutional guidelines standardize blood volumes and no antibiotics were present in the simulation study, individual variation may have influenced retrospective results.

Fourth, simulation CTD measurements lacked continuous recording of growth onset. Because bottles were checked intermittently, precise ITD calculations were not possible, which reduced the resolution and statistical robustness of these data. Furthermore, the small number of strains included in the simulation (*n* = 3 per species) restricted the statistical power of group comparisons; effect sizes and confidence intervals were not reported because estimates based on such small samples would be unstable and potentially misleading.

Fifth, this study used the BD BACTEC™ system. Different blood culture systems may vary in sensitivity, specificity, and growth detection time [28, 29]. Previous comparisons of automated systems, including BACTEC FX, BacT/ALERT 3D and BacT/ALERT VIRTUO, found that VIRTUO detects growth more rapidly, whereas BACTEC FX performs better in the presence of antibiotics [28]. These differences may influence detection of fragile or fastidious bacteria, in addition to detecting *P. aeruginosa* under anaerobic conditions and could affect generalizability to settings using alternative systems.

Finally, the simulation experiments used horse blood rather than human blood. Although horse blood is widely used as a standardized medium, it may not fully replicate human blood’s physiological properties, potentially influencing growth dynamics. Nevertheless, the overall agreement between simulation and clinical findings supports the validity of our conclusions.

## Conclusion

This study, integrating both real-world clinical data and controlled laboratory simulations, demonstrates that minimizing pre-incubation delay is essential for reducing the overall collection-to-detection (CTD) time and preserving the viability of fastidious organisms such as *Streptococcus pneumoniae*. Implementing continuous 24/7 incubation substantially improved CTD in routine diagnostics, while the simulation experiments confirmed the biological vulnerability of certain pathogens to delayed incubation. Together, these findings reinforce the clinical and operational importance of prompt blood culture incubation and support ongoing efforts to optimize pre-analytical workflows in microbiology laboratories.

## Data Availability

The datasets generated and analyzed during the current study are not publicly available due to institutional data protection policies but are available from the corresponding author upon reasonable request and in accordance with Danish law.Requests for access to anonymized data should be directed to **Andreas Jacobs Østerhegn Jensen (email: ** AJEN0809@regionh.dk **).**.

## Abbreviations

BCBs: Blood culture bottles
BSIs: Blood stream infections
CTI: Collection-To-Incubation
ITD: Incubation-To-Detection
CTD: Collection-To-Detection
DCM: Department of clinical microbiology
DCB: Department of clinical biochemistry
MADS: Mikrobiologisk afdeling data system
BD: Becton Dickinson
IQR: Interquartile range
CI: Confidence interval
SD: Standard deviation
CFU: Colony forming units
